# Patient Representation and Advocacy for Alzheimer Disease in Germany and Israel

**DOI:** 10.1007/s11673-018-9871-8

**Published:** 2018-07-31

**Authors:** Silke Schicktanz, Nitzan Rimon-Zarfaty, Aviad Raz, Karin Jongsma

**Affiliations:** 1 Department for Medical Ethics and History of Medicine, University Medical Center Göttingen, Humboldtallee 36, 37073 Goettingen, Germany; 2 Department of Sociology and Anthropology, Ben-Gurion University of The Negev, Beer Sheva, Israel

**Keywords:** Patient organization, Advocacy, Dementia, Representation, Stigma, Disease conception

## Abstract

This paper analyses self-declared aims and representation of dementia patient organizations and advocacy groups (POs) in relation to two recent upheavals: the critique of social stigmatization and biomedical research focusing on prediction. Based on twenty-six semi-structured interviews conducted in 2016–2017 with members, service recipients, and board representatives of POs in Germany and Israel, a comparative analysis was conducted, based on a grounded theory approach, to detect emerging topics within and across the POs and across national contexts. We identified a heterogeneous landscape, with the only Israeli PO focusing strongly on caretakers, whereas in Germany several POs claim to represent this patient collective. Shared aims of all POs were fighting social stigma, balancing the loss of patients’ individual autonomy, and the well-being of caretakers. By highlighting the emergence of new groups of dementia self-advocacy against the more traditional advocacy by others, this study highlights how advocacy and representation in the context of AD are embedded in the discursive context of stigmatization and revised disease conception. Future developments in early diagnosis and prediction of dementia, with more affected people likely to conduct dementia self-advocacy, might challenge existing representation structures even more.

## Introduction

In the last decades, various organizations, associations, and support groups have emerged around chronic patients and people with disabilities as well as their families, mobilizing their “lay expertise” (Epstein 1998) for mutual support, improved health services and access, advocacy, and collaborative research. This article continues and extends previous analyses on such “patient organizations” (POs) by analysing POs *for* and *of* dementia in Germany and Israel. For practical reasons, we use the term “patient organization” as an umbrella concept to encompass a variety of patients/disability advocacy organizations aimed at advocating and promoting the rights and interests of patients/people with disabilities and their family members/caretakers. Organizations *for* such disabilities were established by caretakers or professionals, and organizations *of* were established by and comprised of people with disabilities themselves. This distinction between *of* and *for* is our own conceptualization of a development that has been recognized by others (see, e.g., Gilmour and Brannelly 2010; Moreira et al. 2014).

While previous research on the dynamics of POs and disability advocacy organizations focused on POs involvement in healthcare and research settings (Rabeharisoa et al. 2014; Luce et al. 2011) or on their emancipatory activities (Epstein 1998), we wish to examine these POs as (sometimes conflicting) assemblages of caretakers and patients/people with disabilities in order to gain insights into POs’ claims for empowering collective representation, advocacy, and autonomy. These claims are becoming an important expression of deliberative democracy (e.g., Elster 1998) in the context of neo-liberal managed care and health governance (Dent 2006).

These developments highlight the complex and little-understood issues involved in collective representation in the context of health activism: Does patient advocacy always equal self-advocacy? Should this self-advocacy be prioritized over other forms of advocacy for the patient’s best interest by parents, relatives, caretakers, or doctors? These questions loom large in the context of frailty, vulnerability, and social participation, and continue to present ethical, social, and political challenges for an inclusive society (for a detailed analysis of representative styles in autism POs, see also Raz et al. 2018).

In particular, we scrutinize how the political role and self-understanding of POs are interlinked with the current debate of medical conceptions of Alzheimer’s disease (AD). Dementia advocacy often entails a focus on AD but usually also address other types of dementia. Hence, Alzheimer remains a label in many large POs’ names in Germany and Israel due to historical reasons. Besides, the media refers rarely to other types of dementia (e.g. fronto-temporal or vascular dementia). Dementia, and in particular AD, advocacy offers an interesting topic for investigating the complex interplay of political representation and disease conception for several reasons.

First, AD is an important area where issues of representation and advocacy are pertinent (Golander and Raz 2000). While traditional advocacy for AD is dominated by caretaker relatives, other associations for AD publicly identify themselves as caretakers’ *and* patients’ organizations, reflecting a process of hybridization (O’Donovan et al. 2013). Second, the current debate around AD is characterized by two important upheavals: On the one hand, critical voices impeach the medicalization, stigmatization, and social exclusion related to AD (Werner et al. 2012; Lock 2013; Hazan 2014). On the other hand, biomedical research on the early stages of dementia currently revises the main assumption about the aetiology and trajectory of AD. Alzheimer’s disease is now understood as a continuum and as a slowly progressing syndrome with a long preclinical phase. It is assumed that it starts with an asymptomatic stage and develops into a symptomatic stage involving ‘subjective’ or ‘mild’ cognitive impairment (SCI/ MCI). Eventually it converts into a clinical syndromal disease with an already advanced pathology (Le Couteur et al. 2013; Dubois et al. 2016). Both upheavals impact the scenery of self-advocacy. Alzheimer’s disease advocacy has been dominated by caretakers of AD patients (POs *for*), because patients have been deemed incapable of self-advocacy and self-care. Now, “patients” in the early and even preclinical stages of the disease are increasingly involved in health activism (POs *of*) (Beard 2004).

Furthermore, we employed a national-comparative approach that allows us to reflect upon the impact of the sociocultural framework on this mutual relationship between representation claims and disease conception. For building more general hypotheses, we consequently conducted interviews with representatives of the AD POs’ board and with PO members in Germany and Israel, which are two Western, industrialized countries with social healthcare systems.

### Comparing Germany and Israel in the Context of Alzheimer’s Disease Patient Organizations

Germany and Israel provide a proper basis for this analysis for several reasons. Both countries have issued declarations about the importance of the participation of POs in parliament committee hearings and governmental committee deliberations (Schicktanz and Jordan 2013; Shalev and Hashiloni-Dolev 2011). Furthermore, both countries are at the cutting edge of Western medical progress, with well-developed social healthcare systems. Israel developed a national dementia strategy in 2013, while the German Ministry of Health and Ministry for Social Affairs established an “Alliance for people with dementia” in 2012, which provides a platform to develop strategies and provides information about dementia care. Yet Germany and Israel often represent opposing bioethical regulations and policy processes, in which POs also play a role (Raz and Schicktanz 2016). For example, in end-of-life decisions, Germany is rather permissive in comparison to Israel. It allows passive euthanasia if patients wish it and requires doctors to follow advance directives quite strictly, independently of how and when they have been composed. In Israel, passive euthanasia is allowed under very strict conditions, and advance directives are strictly regulated regarding format and registration (Schicktanz et al. 2010). However, in beginning-of-life healthcare, such as prenatal genetic testing and reproductive medicine, Israel is very permissive in comparison to Germany (Raz and Schicktanz 2009).

In both countries, disability politics and activism are based on the expansion of welfare and special needs provisions while the social movement for equal rights and integration followed later (inspired by the U.S. disability debate). Additionally, in both Germany and Israel, there is a (quite similar) Disability Act; but while in Germany the disability movement appears to have formed a mature coalition (Heyer 2002), in Israel there is no such coalition and the disability community is divided between sectors (disabled veterans/workplace disability/general disability), advocacy and service organizations, and even between competing organizations for political activism of people with disabilities (Gilad and Rimmerman 2014; Mor 2009). Similar to other European countries, in the last five years dementia has gained more attention and public awareness than ever before in both Germany and Israel, giving the impression of a “dementia hype” (Swinnen and Schweda 2015).

### Research Questions and Main Objectives

Starting from these general political-ethical issues we want to examine *how advocacy and representation in the context of AD are embedded in the discursive context of stigmatization and revised disease conception*. The comparative empirical analysis aims at detecting cross-cultural variances in debates and more general discursive structures otherwise easily overseen in local studies. In our analysis, we subsequently present similarities and differences in how German and Israeli POs *of* and *for* AD patients describe their practical and political aims, as well as their practices and positions towards representation. Moreover, we embed these findings in the current debates on stigmatization and pre-clinical dementia diagnosis. Finally, we conclude with some general reflections on the question of how representation is relevant for addressing ethical issues of patient involvement and collective decision-making in healthcare.

## Methodology

The first stage of this study consisted of background analysis of guidelines, policies, websites, and reports regarding the status and involvement of dementia POs in Germany (GE) and Israel (IL) (for an additional comparative analysis of Germany and the United States, see also Schicktanz 2017). In Israel only one large PO exists *for* people with AD. EMDA—the Alzheimer’s Association of Israel—was established in 1988 by family members as a nationwide association. It consists of about five hundred official members and provides service to 30,000 to 40,000 Israeli AD patients and family members. These numbers are based on EMDA’s board member assessment as reported in a personal interview. Another non-profit, service-operating association is Melabev. This geriatric specialists’ association focuses on care and service provision and less on advocacy and was therefore not included in our analysis. In Germany we identified various associations. The German Alzheimer Association is the largest umbrella organization *for* AD patients, consisting of about 15,000 official members in about 500 regional support groups. In addition, several small associations exist. Based on the background analysis, in each country we approached the politically most active and relevant POs. Each PO established a board consisting of one director (or so-called chairs) and various office holders. This board is usually responsible for decision-making and communication. Directors of these POs were contacted directly via email. Additional chairs and other board members were recruited using snowball-sampling via the directors. Professional contact from former academic workshops existed in two cases, but no personal contact between researchers and the interviewed representatives existed before. We approached additional smaller associations *for* and *of* AD patients on the basis of their public outreach and included therefore Alzheimer Ethics and Dementia Support Stuttgart (POs *of*), as well as a regional office of the umbrella organization and Action Dementia (POs *for*) into our sample (see table 1). In German POs *of*, people with dementia are directly involved in establishing or operating self-representation and advocacy. Overall, in 2016–2017 we conducted fourteen interviews in Germany and twelve in Israel. Overall, we interviewed three directors, two chairpersons, five board members, three other office holders, three members with AD, and ten caretakers members/service recipients. Ten of participants were male and sixteen were female (see table 2). The interview collection was finalized when the research teams agreed that thematic saturation was reached, meaning that no new topics were raised in the subsequent interviews. In Germany, this took two more interviews. Research ethics approval was received from the Research Ethics Committees of Ben-Gurion University (Israel) and of University Medical Center Göttingen (Germany).

**Table 1:** Overview and background details of all included POs in Germany and Israel

**Organization** ^**1**^	**Year of establishment**	**Organizational structure & membership composition**	**Membership size**	**Goals**	**Involvement in health policy**	**View on condition** (quote from the website)
**Deutsche Alzheimer Gesellschaft e. V.** ***(for)*** Umbrella association (138 regional organizations), registered association	1989	**Board of directors; advisory board with people with dementia** is convened Services to: Patients with dementia, relatives, caretakers, medical professionals	ca. 15.000	Increase understanding of dementia. Improve self-managernent of relatives. Support scientific research regarding dementia and develop new forms of care-taking.	Involved in **political lobbying** and **political decision-making. Participate in committees and assigned by the ministry of health for legislative processes**, e.g. care support law.	Biomedical: “Even when it is not dementia, in many of these cases medical treatment or therapeutic support is indicated.”
**Demenz Support Stuttgart GmbH** ***(of)*** Non-profit private limited liability company, funded by Erich and Liselotte Gradmann foundation	2002	**Board of directors; advisory board all 5 advisors have dementia);** employees (8-12); team of curators Services to: Patients, care-takers	-	To promote the well-being of people with dementia and their care-takers and to realize their participation in society. Mediate between science and practice. Foster exchange among different disciplines.	**Involved in negotiations with ministries** in legislative processes on new residence and participation law.	Societal challenge: „Dementia is a social and cultural challenge for society. [..], people with dementia need good nursing and medical care. But they need more than that: a social environment in which they are welcome and, of course, part of.”
**Alzheimer Gesellschaft München e. V.** ***(for)*** Regional association, registered association	1986	**Board of directors** (1 director, 5 committee members); 50% relatives in general assembly Services to: Patients, relatives	ca. 400	To represent the interests of people with dementia, their relatives and care-takers and offer counseling. Promote self-help potential within families and friends.	Participate in committees and **working groups** and cultivate contacts on the **local level to** promote care for people with dementia.	Biomedical:“is understood as disturbances of mental achievements of the memory, the thinking and orientation property as well as the language. Due to these disturbances, coping with everyday life becomes more difficult.”
**Alzheimer Ethik e. V.** ***(of)*** Registered association	2000	**Board of directors** (2 members); **extended board** (3 representatives of active members; people with dementia) Services to: Patients, relatives	ca. 200-500	To support the dignity and quality of life of people with dementia. To inform patients and family members on non-pharmaceutical treatments. Pose ethical questions concerning dementia.	Maintain **contacts with politicians** in. health sector.	Societal: „People diagnosed with dementia belong in our society. They should not be isolated in homes or other institutions.”
**Aktion Demenz e. V.** ***(for)*** Registered association, funded by Robert-Bosch foundation	2006	**Board of directors** (l0 members) Services to: Society	ca. 50	Support civil engagement to improve living conditions of people with dementia. Aim to diminish stigmatization by raising awareness. Inform the public and experts.	**No direct involvement,** but they influence the political lobby by strengthen the idea that social action is of utmost importance.	Biomedical: “Dementia implies increasing destruction of intellectual capacities and personality. An independent everyday life without . help from others is clearly impaired or even irnpossible.”
**EMDA—the association for patients with dementia, Alzheimer and similar diseases in Israel** ***(for)***	1988	**Board of directors** (12 members) elected by the **general assembly**. Alongside the Board is the **scientific committee** whose members are leading medical, legal. and nursing professionals The association operates a separate **critic committee** Services to: Patients, relatives, care-givers and professionals	ca. 500 (But is estimated to provide services to 30.000-40.000 patients and relatives)	To create a network for families of people with dementia and offer them information and emotional support. Develop support services for people with dementia and their relatives. Raising public awareness and knowledge about dementia, and the rights of people with dementia and their relatives. Promote research into dementia.	Involved in **political lobbying** to promote the rights of people with dementia and their relatives. **Cooperation with the Ministry of Welfare, the Ministry of Health (the geriatric ward) and the National Insurance Institute.**	Incurable biomedical condition: "With the increase in life expectancy and the aging of the population, dementia has become a modern-age epidemic. Dementia is characterized by a decline in intellectual functioning and memory abilities, changes in behavior, and difficulty in performing daily activities"

^1^Organization names are translated into English, by providing the original name (e.V. = is German registered non-profit society; gGMBH = charitable limited company)

**Table 2:** Overview of interviewees with POs in Germany and Israel

Patient Organization	board members and office holders	Member, with AD	member, family caretaker	Total N
**Germany**				14
**German Alzheimer Association** **(** *Deutsche Alzheimer Gesellschaft e. V* **.)**	1		1	2
**Alzheimer Ethics** **(** *Alzheimer Ethik e. V.* **)**	1	1	3	5
**Dementia Support Stuttgart** **(** *Demenz Support Stuttgart gGmbH* **)**	1	1		2
**Alzheimer Association Munich** **(** *Alzheimer Gesellschaft München e. V.* **)**	1	1	1	3
**Action Dementia** **(**Aktion Demenz e. V**.)**	1		1	2
**Israel**				12
**EMDA- the Alzheimer's Association of Israel** **(** ***בישראל דומות ומחלות אלצהיימר דמנציה לחולי עמדא עמותת*** **)**	8		4	12

All interviews were semi-structured with similar questions asked in Germany and Israel (see the questionnaire for board members/office holders and regular members/service recipients in the supplementary material). Office holders were asked about the history of the organization, their activities, and the organizational goals, successes, and failures, relation to other associations, positions towards biomedical research, and the internal structures of decision-making, including the association’s policies regarding the adequate representation of the range of voices present within its constituency. Association members were asked about their involvement in PO activities, to what extent they are involved in PO decision-making, how satisfied they are with it, and who should be involved in organizational decision-making. They were also asked about their position and activities towards care in dementia and towards ethical-legal debates. Interviewees were also asked about their personal link to AD advocacy (e.g., whether they were caretakers or diagnosed with dementia). Both were considered as phenomenologically “being affected” and also addressed in these terms in our interviews, in contrast to professional office holders who have had no personal involvement (non-affected). Interviews were conducted in the office or home of the respondents or over the telephone (when preferred by the interviewee) and lasted between thirty and ninety minutes. Association members/service recipients were recruited via office holders, the associations’ website, and the associations’ support groups; we used the snowball method to reach additional members. Consent was obtained from all participants. Interviews were digitally recorded and verbally transcribed. Transcripts were translated into English and anonymized, with the POs’ names replaced by a code, PO1 to PO5. Using a grounded theory approach to data analysis (Corbin and Strauss 1990), emergent themes concerning perceptions and self-declared aims of the PO were identified through inductive coding using ATLAS.ti©. Interviews were first analysed thematically in each country and compared cross-nationally by the research team to uncover discursive themes connecting general representation styles found in the literature on disability advocacy with the particular context of AD, looking for themes and their variations recurring within and across organization type and country (Denzin and Lincoln 1994).

## Results

Our analysis revealed the following three common topics. These are broadly shared and uncontroversial for Alzheimer advocacy in both countries: *fighting social stigma, balancing the loss of individual autonomy, and the well-being of family caretaker* (see table 3).

**Table 3:** Overview of topics that emerged from the interviews with POs board members and constituency members in GE and IL

	**German POs**	**Israeli PO**
***Of/for*** **: Shared topics**	• **Fighting social stigma:** Social acceptance as human being/citizen; treatment to improve conditions • **Balancing the loss of individual autonomy:** Collective voice for claiming individual needs in social and medical care; heteronomy as normality; fighting for legal opportunities to extend autonomy (supported decision-making as alternative to traditional guardianship, advanced directives) • **The well-being of family caretakers**: In need of care themselves; but risk of overlooking the patient • **Biomedical model of dementia**	
***For***	• **Public awareness of dementia**: Holistic approach in social/medical care (favouring dementia friendly community approaches) • **Representative political practice:** Trustee model (competence more important than having the disease) • **Intra-family relationships:** Family members idealized as trustees/no alternative	
***For***	• **Representation style:** More open to self-advocacy, but prefer a complementary approach	• **Representation style:** Very critical towards self-advocacy, insisting on caretakers or volunteers
***Of***	• **Public awareness of dementia**: Stressing agency and independency of patients (favoring assistive technologies) • **Representation style:** Ideal of self-representation/delegate model • **Intra-family relationships:** Mentioning conflicting interests between patients and care takers	N/A

### Fighting Social Stigma

Overall, our interviewees in both countries stressed that according to their perception, dementia is a “special” illness, highly associated with *social stigmatization* because of the progressive and irreversible influence on cognition that affects the personality of the person with dementia. Interviewees highlighted the differences between AD and other (now) less stigmatized illnesses such as cancer. This point was brought up by several interviewees.

The only explanation I have is that there is no cure for this disease. It is impossible to beat the disease. This disease usually does not harm children or young people. It is not a disease such as cancer, with heroic cases, that you can win it over and be a hero. So apart from suffering and shame, it is not accompanied by anything. And the public is not making his [the patient] voice heard. They are becoming more and more introverted and try to survive. (PO *for*, board member, Israel)

Overall, fighting stigma was perceived as a main goal for all the POs we studied, as demonstrated by the following quote of an interviewee with AD:

I do not want to be pathologized—I want to be seen as a human being and I want to be asked accordingly. In the consultation, I expect to be treated a certain way, that means the language they use when talking to me. It should not be a language, for example, that goes as follows: “you are suffering, you poor, your mind is dwindling every hour, this deprivation, this perplexity. How do you fight these symptoms?” This is a language that shows me that they pathologize me and— they do not provide support, they take all away from us.” (PO2 *of*, member with dementia, Germany)

The problem of social stigma led some office holders to mention their struggle to recruit new members, because being associated with the PO may exacerbate the member’s stigma.

Overall, both POs, *of* and *for,* supported the biomedical model of dementia. Many POs provided a platform for recruiting potential research participants. The hope was that with better treatment and care, stigma and social exclusion will diminish. Therefore, involvement in, and collaboration with, scientific research, including biomedical research, was regarded positively, even though the POs’ own direct financial investments in biomedical research were limited. In the case of the German Alzheimer Association, their funding of research was related to improving care, housing, or communication. In Israel, EMDA did not directly support or fund any kind of research but provided links to existing research for participation. However, many interviewees criticized the national policy for focusing too much on medical and hospital treatment and for setting very limiting criteria for social security support, and thereby overlooking the needs for specialized social support for caretakers as well as for persons with dementia outside the hospital context.

### Autonomy and the Caretaker

Another theme shared by all interviewed PO members was the effect of the diagnosis of dementia on the *autonomy* of the diagnosed person. First, a diagnosis may lead to heteronomy, that is, being governed by third parties (e.g. family members gain the power to decide in minor and major life decisions, including decisions over driving, mobility, housing, and financial issues). A typical comment was:

[people with dementia] experience this as something very bad when they say: “As soon as I have this dementia … in the sense of the diagnosis … my relatives do not take me seriously anymore, suddenly decide all things, therefore terribly restricting my freedom.” This must be clarified from the beginning that too much of this approach under the notion of care—“It’s me who decides now, I do not believe you are able anymore”—it is very, very discriminating for the person affected, I believe, that this makes him more incapacitated … (PO3 *of*, board member, Germany)

Secondly, since AD is understood as a progressive neurodegenerative disease that will result in loss of memory, planning, orientation, and assessment, it makes the interpretation of the patient’s wishes more challenging:

Sometimes the words of a person with dementia come from the dementia and not from himself. (PO *for*, member, caretaker and service recipient, Israel)

POs try to balance this loss of individual autonomy in several ways. According to their understanding, the collective voice replaces the individual voice in expressing needs and interests about care and treatments. Additionally, the POs engage in political debates in each country about advanced care directives or guardianship. This engagement can also be interpreted as “extension” of the autonomous individual voice (especially against the paternalism of professionals) to ensure (more) self-determination of the person with dementia. However, there remains an ambiguity in this position, as the POs *for* strongly favour the family caretaker as the best proxy for decision-making.

Alzheimer’s disease was framed as a fatal disease characterized by loss of accountability and therefore mainly associated with obvious, already developed symptoms. The few active members with dementia were characterized as fitting into the category of very early stage of dementia. The topical issue of predictive testing of prodromal dementia was not mentioned by the interviewees.

It was expressed as typical of the condition that both patients and their families (as caretakers) are phenomenologically “affected by” AD. In contrast to other chronic diseases such as cancer or HIV where family members are rarely mentioned as an affected group, for dementia this was different. Family members who act as caretakers are seen as almost similarly affected:

… although we always argue, dementia is actually a disease of the relatives because they suffer quite a lot. (PO1 *for*, relative and caretaker, member, Germany)

We have activities aimed to give patients quality of life, but we are more directed to providing the tools to enable caregivers and help them survive the process. (PO *for*, board member, Israel)

This focus on the caretaker entails the risk that the diagnosed person herself is not given the necessary attention anymore or that the burden of, and concerns for, the family caretakers are overemphasized. Some German interviewees stressed therefore the need to organize separate activities for caretakers and for people with dementia.

### Understanding Dementia in POs *Of* and *For*

Alongside these topical overlaps, we also identified important nuances in themes and opinions between POs *for*—both in Israel and Germany—versus the German POs *of* (see table 2). These differences can also be classified along the three themes of 1) public awareness of dementia, 2) representative political practice, and 3) intra-family relationships.

Regarding *public awareness of dementia*, POs *for* and POs *of* differed in the detailed characterization of how dementia should be framed. Germany and Israel POs *for* stressed a rather “holistic” approach of medical *and* social support for all affected persons, including patients and their relatives as caretakers. They also called to fight the tabooing of the disease as such and to provide comprehensive information for lay people about the progressive course of the disease.

… to get the subject a bit out of the taboo zone. This is very important to me, the more you know about it, the less fear you need to have. I do not want to play the topic down, and it is certainly, when one has a diagnosis, primarily for the affected person and of course for the family members certainly incredibly devastating. But there is a life after that. (PO5 *for*, relative and caretaker, member, Germany)

The POs *of* aimed at—literally—moving into society to raise awareness, showing their autonomy and agency despite the condition, by exposing the public to people with early stage dementia. Members with early stage dementia initiated several projects raising public awareness about the existing social mechanisms of exclusion and the fight for normalization—especially for patients in the early phases of dementia. Such activities included visits to schools and public institutions.

One German interviewee of a PO *for* stressed that normalization needs to be embedded in a dementia-friendly community and to allow ambulant support as long as possible:

So the goal is to keep living independently as long as possible, so in his own living environment and it starts, for example, that a household assistance is necessary (…). And then one gradually builds up the supply structure with some sort of shifts so that the relatives are relieved hourly (…) to a certain point, when it is perhaps not good any more to live alone at home, but it is then simply important that you move into a facility. (PO4 *for*, board member, Germany)

In slight contrast, the POs *of* stressed particular assistive technology for ensuring independence. As the quote indicates, the idea that sooner or later a care home will be the logical end, is yet not so self-evident.

Very important topics are, for example, assistive technology. When I feel worse, how I can help myself, yes, how I can help myself remain to live at home independently. (PO2 *of*, member with dementia, Germany)

While we gained the impression that POs *of* do benefit practically from members and representatives being diagnosed early, neither them nor PO *for* expressed a clinical/medical interest in early detection of dementia via biomarkers or genetic testing.

The difference between national neuro-cultures and self-advocacy became most visible in the understanding and practice of representation. At one end of the spectrum was the Israeli PO *for* which was not ready to have self-advocacy at all:

It fits American political correctness, but elsewhere? Then I ask you—what are they useful for? What contribution do they have to make? What can they contribute? I do not have a concrete example of such a contribution. I’m sceptical about that. (PO *for*, board member—non-caretaker, Israel)

The German POs *for* were more positive about self-advocacy (some included persons with dementia in their advisory board) but defended a mixed, complementary approach:

The good thing is that nowadays the people with a dementia diagnosis can be diagnosed very early (…) and if you talk to the people who are early-affected, who are also able to express themselves, then they clearly say, “we speak here with a loud voice, but we also speak for the people who may already not have strong words, who do not find the words anymore.” (PO4 *for*, board member, Germany)

In contrast, POs *of* stressed an ideal of self-representation:

… it was not a topic at all here in Germany until at least some years ago (…). It was observed with disbelief and not understood what we actually aimed for; how we come to believe that those who are affected by dementia can actually think or articulate themselves. (PO3 *of*, board member, Germany)

Interviewees from POs *for* did not ignore potential conflicts that arise when caretakers and relatives speak on behalf of the patient. However, they either stressed the incapacity of the patients or the burden of caretakers:

Their voice is always through the family, unless there is a direct connection between the volunteer [*an unpaid helper trained by the PO, the authors*] and the patient, which is also usually a surprise to the family, whether it’s in terms of its capabilities, whether it’s things that the family thinks that he stopped doing and suddenly they discover that with the volunteer he still does. Often the volunteer comes in, usually in the early stages of the disease, and the patient is still less expressing himself, but a family member is expressing his wishes for him! So there it is. So really our volunteers are our ambassadors in the field. (PO *for*, office holder 3, Israel)

In contrast, interviewees from POs *of* explicitly and critically reflected upon problems with family members that even led to intra-familial discrimination. For them, it was important that family members acknowledge the person with the resources they still have. Interviewees from POs *for* also pointed out the emphasis on expectations regarding the *competences* of the caretaker (as attentive, trustful, creative, etc.). Interviewees from POs *for* tended to focus only on social discrimination and located the problem in the psychological denial of persons with dementia to recognize their limitations. Importantly, from a cross-cultural perspective, and though holding different positions in the context of intra-familial relationships, both POs *of* and *for* in Germany shared a resource-oriented rather than deficit-oriented approach.

## Discussion

Our findings highlight how beyond the shared public and political goals of AD advocacy, Germany has a more diverse landscape of AD organizations including organizations *of* people with early-stage dementia, in contrast to a more uniform landscape in Israel. By highlighting the emergence of dementia self-advocacy against the more traditional backdrop of advocacy by others, this study highlights how advocacy and representation in the context of AD are embedded in the discursive context of stigmatization and revised disease conception. In the Israeli PO *for*, we found a strong focus on caretakers in a double sense: they are considered competent to represent the patients’ best interest, and the service provided by the PO is aimed at reducing the burden of caretakers. Here, the leading idea is that helping the caretaker will eventually help the patient. Scepticism towards self-representation of people with dementia is based on the assumption that dementia is related to denial and loss of self-identity.

However, the lack of self-representation of people with AD might increase the social stigma as it fuels existing stereotypes of helplessness, identity loss, and incapacity (Beard 2004). In political-ethical terms, the issue of self-representation is less a question of efficacy of interest presentation than of political self-determination (Williams 1998). The misrecognition of the right of people with AD to represent themselves may be internalized by them and further harm their self-esteem and sense of agency, which may lead to the reinforcement of hierarchies impeding equal participation (Williams 2014).

In Germany we observed a larger pluralism regarding representative structures as they exist now in POs *of* and *for*. This plurality leads to a more explicit recognition of the inherent limits of representation-styles and has created a discursive space of critical but friendly interaction. However, one needs to be realistic that the well-established and politically better-integrated POs *for* leave only little room in the political landscape to be filled by upcoming POs *of*. In other areas, such as autism, a split of POs *of* and *for* results in POs *of* often questioning their status as patients by understanding themselves as “neurodiverse,” but not as ill or pathologic (Raz et al. 2018; Chamak 2008). In the context of dementia POs, however, such radical split with regard to political aims and conceptualization of the condition is absent. One explanation may be found in the existing social stigmatization of dementia and insufficient healthcare structures for dementia care. These two social obstacles form a joint, unquestioned premise for both POs *of* and *for* and unify them in their advocacy aims. However, it can be argued that under such conditions of social stigma, which stress and amplify the loss of the patient’s self-determination, agency, and autonomy, the new emergence of self-advocacy dementia groups is in itself *radical*. The rather passive, yet positive, attitude towards biomedical research of POs in Germany and Israel differs from the American Alzheimer Association, which is a leading player in biomedical and pharmaceutical research as well as in neuro-imaging and predictive testing of dementia, especially AD. The main focus of the POs in Germany and Israel is rather on fighting social stigma by improving social and community structures. The occurrence of dementia self-advocacy in Germany but not in Israel indicates the cultural entanglement of stigma and advocacy in the context of dementia. It even highlights a problematic ambiguity when POs *for* address the stigma of dementia especially as part of fundraising campaigns that capitalize on vulnerability, but by this, they may inadvertently amplify it. Many of the Israeli office-holders of the PO *for* stressed that stigma was the reason for the organizational agenda of raising awareness, as well as the explanation why early-stage dementia patients were not ready to pay the social price of being labelled as persons with dementia, thus avoiding the PO’s services. It appears that POs *of,* such as those in Germany*,* have a crucial role to play in breaking this vicious circle of stigma of dementia.

Overall, a general ethical issue from a theoretical perspective remains as to how the patient perspective can be represented in a balanced and consistent way in healthcare politics: The possibility of *self*-advocacy warrants assistance and support, even if the relatively newer and smaller POs *of* dementia that are emerging in Germany have not yet differentiated themselves so much, in terms of what they are lobbying for, from the POs *for*. In comparison to Alzheimer Europe and Alzheimer US and their focus on “expert” and professional voices, the integration of early diagnosed patients or high-functioning patients in advocacy structures can be understood as convergence and might strengthen the existing PO *for*. For example, the umbrella organization Alzheimer Europe started to stress the positive potentials of early diagnosis for preparing advance care planning and advance research directives,^1^ but is very hesitant to discuss the concrete potential for its own practice of political representation. Future research on these debates and negotiations could help to identify the limits of scepticism or political interests and foster a critical discussion. In this sense, we speculate that current developments in dementia diagnostics and prediction will likely lead to more interesting changes in the structures of POs in case the POs are overall open for these developments.

## Limitations of the Study

The small sample in our study (twelve interviews in Israel and fourteen interviews in Germany) limits the claim for generalization of our findings. The main aim of our study was to explore working hypotheses regarding the diverse aims and approaches of collective representation in Alzheimer POs, to describe and reflect on the normative implications of and cultural impacts on the division between POs *of* and *for*. Examining dementia POs in other countries would be helpful to understand how our observations are part of a larger cross-cultural landscape in dementia advocacy.

## Footnotes

1
http://www.alzheimer-europe.org/Policy-in-Practice2/Our-opinion-on/Advance-directives
